# Inter-rater reliability of categorical versus continuous scoring of fish vitality: Does it affect the utility of the reflex action mortality predictor (RAMP) approach?

**DOI:** 10.1371/journal.pone.0179092

**Published:** 2017-07-13

**Authors:** Pieter Meeremans, Noëlle Yochum, Marc Kochzius, Bart Ampe, Frank A. M. Tuyttens, Sven Sebastian Uhlmann

**Affiliations:** 1 Vrije Universiteit Brussel (VUB), Brussels, Belgium; 2 Flanders Research Institute for Agriculture, Fisheries and Food, Animal Sciences Unit, Ostend, Belgium; 3 Oregon State University, Corvallis, Oregon, United States of America; 4 Faculty of Veterinary Medicine, Ghent University, Merelbeke, Belgium; University of Tasmania, AUSTRALIA

## Abstract

Scoring reflex responsiveness and injury of aquatic organisms has gained popularity as predictors of discard survival. Given this method relies upon the individual interpretation of scoring criteria, an evaluation of its robustness is done here to test whether protocol-instructed, multiple raters with diverse backgrounds (research scientist, technician, and student) are able to produce similar or the same reflex and injury score for one of the same flatfish (European plaice, *Pleuronectes platessa*) after experiencing commercial fishing stressors. Inter-rater reliability for three raters was assessed by using a 3-point categorical scale (‘absent’, ‘weak’, ‘strong’) and a tagged visual analogue continuous scale (tVAS, a 10 cm bar split in three labelled sections: 0 for ‘absent’, ‘weak’, ‘moderate’, and ‘strong’) for six reflex responses, and a 4-point scale for four injury types. Plaice (*n* = 304) were sampled from 17 research beam-trawl deployments during four trips. Fleiss kappa (categorical scores) and intra-class correlation coefficients (ICC, continuous scores) indicated variable inter-rater agreement by reflex type (ranging between 0.55 and 0.88, and 67% and 91% for Fleiss kappa and ICC, respectively), with least agreement among raters on extent of injury (Fleiss kappa between 0.08 and 0.27). Despite differences among raters, which did not significantly influence the relationship between impairment and predicted survival, combining categorical reflex and injury scores always produced a close relationship of such vitality indices and observed delayed mortality. The use of the continuous scale did not improve fit of these models compared with using the reflex impairment index based on categorical scores. Given these findings, we recommend using a 3-point categorical over a continuous scale. We also determined that training rather than experience of raters minimised inter-rater differences. Our results suggest that cost-efficient reflex impairment and injury scoring may be considered a robust technique to evaluate lethal stress and damage of this flatfish species on-board commercial beam-trawl vessels.

## Introduction

Observer rating of an animal’s condition (e.g. visually evaluating physical, physiological, and behavioural states) is an established tool in animal welfare research to integrate multi-modal information and describe complex behaviours, which may be difficult to measure otherwise [1]. In fisheries science, to evaluate vitality and extent of injury of whole animals, the condition status of captured organisms can be assessed by observers (here ‘raters’). Their ratings involve abstraction and interpretation of criteria, which might be influenced by experience, cognitive ability, and other personal traits, and hence might be biased [1, 2]. The effect of such inter-rater variability on the application of the ratings has to be quantified, before any index can be recommended [1, 3–4]. This is especially true if many raters become involved and their data are pooled [5–6]. Nevertheless, despite differences among rater’s scores, inter-rater reliability might still be high, if, for example, scoring differences are consistent [2].

Examining an animal’s health might be preferable to physiological samples of blood or plasma for reasons of cost-efficiency and feasibility (e.g. harsh conditions on-board moving vessels may prevent the use of expensive, sensitive, or invasive measuring equipment) [6–10]. Condition status has been characterised in the field by scoring the extent and severity of injury, such as epithelial damage to the scales [11–12] and skin on an ordinal scale (catch damage index) [13–14]. For example, multifocal petechial haemorrhages and suffusion of the head or body region were strong predictors of delayed mortality of European plaice (*Pleuronectes platessa*) [6,14]. While injuries reflect external physical impact, responsiveness to induced stimuli expressed as a binary presence-absence score, mirrors internal physiological stress responses (‘reflex action mortality predictor’, RAMP) [7, 15–16]. Reflexes are fixed, innate action patterns independent of sex, size, or motivation [8]. Reflex impairment can be induced by many types of stressors, including exhaustion, hypoxia, or increased temperature. These stressors alter neural and muscle systems that compose reflex actions. The pathway of nerve impulses from the receptors to the muscles through the brainstem and/or the spinal cord [17] might be affected by stress via an altered metabolism from anaerobic exercise and hypoxia which in turn may lead to impaired reflexes [7, 18–20].

Impairment can be expressed as a simple proportion of impaired reflexes, or reflexes, and injury scores to generate a vitality index[7]. Such indices can be related to a mortality probability gleaned from tagging, or captive holding observation. Such a RAMP relationship links mortality to reflex impairment (and injury) [7, 16, 21–22]. The RAMP method is gaining popularity for providing a proxy for mortality attributed to fishing stressors [8, 10, 21, 23–24] due to its more direct link between interacting stressors and mortality than traditional physiological metrics, such as blood plasma cortisol, lactate, glucose and ion concentrations [8, 25].

However, such reflex impairment and injury scores could be prone to subjective judgement [7, 26–28] and lead to bias, if raters were influenced, for example, by their own expectations about an outcome given their knowledge of a certain treatment. Bias may also be introduced by classifying body movements as either present or absent, while in fact, at least some reflexes (such as swimming and breathing movements) might be observable as part of a continuous response spectrum varying in speed, intensity, or frequency [1, 29]. For example, scoring body movement might make it difficult to decide whether a weak movement is now truly absent or not. Doubt about the presence of a reflex or a weak response might incline some observers to call it either absent [”rule of doubt” suggested and practiced by 7, 24–26], or present (performed as part of the data analysis of this study). Except for [30], the value of adding a “weak” category to the binary absence/presence scoring scale has not been thoroughly evaluated yet. In their work on Bering Sea crustaceans, [30] tested how adding a “weak” category for a reflex response alters logistic regression results, if reflex impairment scores were calculated by either including or excluding “weak” with scores for absent responses. In contrast, the advantage of using a binary presence-absence compared to a multi-level scale is, that scoring criteria are more readily memorised by practitioners in the field and less overlap might exist between them [31]. Alternatively, reflex actions might be scored on a continuous scale to capture all nuances of a response’s intensity and speed [32–33] and to increase power of statistical inference when describing vitality-mortality relationships [34].

Based on the logic above, in this study we aimed to evaluate (i) whether different raters are able to reproduce scores for reflex impairment and injury of the same fish, even if different scoring scales were used (categorical vs continuous); and (ii) whether the statistical relationship between indices and delayed mortality was influenced, either by which rater scored or which scale was used for scoring.

## Materials and methods

### Ethics statement

This research was approved by the animal ethics commission of the Flanders Research Institute for Agriculture, Fisheries and Food (ILVO, Ref. no. 2016/264). Experiments were performed on-board the R/V *Simon Stevin* and at a research laboratory in Ostend, Belgium.

### Equipment and treatments

During four day-trips aboard the R/V *Simon Stevin* in the southern North Sea (51°22’N, 02°04’E) in December 2015 and February/March 2016, either a 3-m beam shrimp trawl with a 20-mm mesh codend (equipped with a tickler chain, trips 1, 2 and 4; Table 1), or a 4-m beam chain-mat flatfish trawl with a 80-mm mesh codend and a benthic release panel were deployed for < 60 min (trip 3; Table 1). The gear and deployment duration were typical of the Belgian coastal beam-trawl fishery for common sole (*Solea solea*) or brown shrimp (*Crangon crangon*). From four or five deployments during each trip, 20 plaice (within the size spectrum of commercially caught-and-discarded fish; see [6]) were randomly picked from the catch when the codend was emptied on deck. These fish were placed in batches of five into 50-L, dry baskets. For each collected fish, the time between it being landed on deck and the start of reflex and injury assessment was measured as air exposure duration (in min). To control for the effects of researcher-related handling (reflex and injury assessments, length measurement and tagging, as described below), on each trip, ten plaice were taken from aquaria in Ostend and put into similar holding containers as the study fish following [6]. These fish were not exposed to any additional stress from trawl capture and handling.

**Table 1:** Summary of technical, environmental, and biological variables (Mean ± SD, where applicable) recorded during four day trips with the RV *Simon Stevin* in the southern North Sea.

Variable	Trip 1	Trip 2	Trip 3	Trip 4
Trip date (MM/DD/YY)	12/17/15	02/18/16	02/26/16	03/10/16
Latitude / Longitude	51°24’N, 02°04’E	51°26’N, 02°08’E	51°33’N, 02°06’E	51°36’N, 02°08’E
Beam length (m)	3	3	4	3
Deployments sampled	5	4	4	4
No. of plaice sampled	64	81	80	79
No. of control plaice	0	10	10	10
Sampled plaice total length (TL, cm)	18 ± 3	23 ± 5	22 ± 4	20 ± 6
Trawl duration (min)	43 ± 6	45 ± 0	38 ± 8	46 ± 1

### Reflex and injury assessments

**Fig 1:**
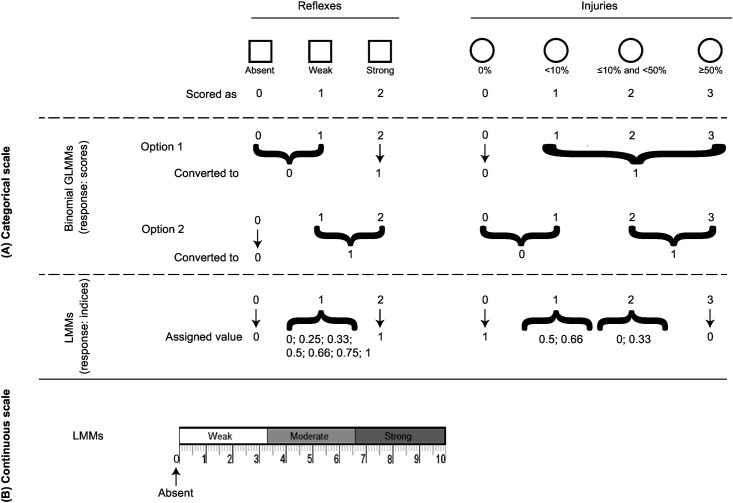
Categorical (A) and continuous (B) scales for scoring reflex responses and injury. The categorical scale to score reflex responses comprised a 3-point scale: absent:0; weak: 1; and strong:2) and to score injury comprised a 4-point scale: 0:absence of injury; 1:< 10% surface area coverage; 2:between ≤ 10% and < 50%; and 3—≥ 50%). The continuous tagged analogue visual scale (tVAS) consisted of a 10 cm bar split into three equally sized 3.3-cm sections, whereby 0 represented absent, and > 0 and ≤ 3.3 cm was labelled as ‘weak’; > 3.3 and ≤ 6.6 cm as ‘moderate’; and > 6.6 cm as ‘strong’. To test for any inter-rater differences of categorical reflex and injury scores, binomial generalised mixed effects models (GLMMs) were fitted (A). For the binomial GLMMs, categorical reflex or injury scores were turned into binary variables according to two options. Option 1 considered weak reflexes to be scored as absent, and slight injuries as present (weak fish may have been in fact weaker and more injured than apparent); or option 2 considered weak reflexes to be scored as strong, and slight injury as absent (weak fish may have been in fact more lively and less injured than apparent). To test for the magnitude of inter-rater differences of the continuous scores, linear mixed-effects models were used (B).

To test for inter-rater differences, three raters scored each sampled plaice simultaneously, but independently, for reflex responses and bleeding injuries using a scoring sheet with a layout which included two scales (both a 3-point categorical and continuous scale for each reflex, and a 4-point categorical scale for each injury type; Fig 1). Rater A was a fisheries research scientist who developed the scoring criteria as shown in Table 2, and who, prior to this study, scored 45 flatfishes for reflex impairment (plus an additional 215 fish were observed being scored by a colleague); rater B was a technical fisheries research assistant who had previously collected vitality data on-board commercial vessels for 998 flatfishes (940 additional observed); and rater C was a fisheries science student for whom the reflex testing concepts were novel and training only consisted of scoring 26 flatfishes himself (37 additional observed). None of the raters had experience with a 3-point categorical nor a tagged visual analogue scale, termed ‘tVAS’ for scoring reflexes. A categorical scale was used for scoring in addition to a continuous scale (Fig 1). The categorical scale consisted of three categories (‘absent’: scored as 0, ‘weak’: 1 and ‘strong’: 2), and the tVAS scale a value from 1–10, indicated on a 10 cm bar, split into three–equally sized 3.3 cm sections, labelled ‘weak’, ‘moderate’, and ‘strong’ (Fig 1). The categorical scale used for scoring injuries of the head and body (multifocal cutaneous petechiae—hereafter termed ‘point bleeding’, and suffusion or haemorrhaging—termed ‘bruising’) included four categories: 0 –absence of injury; 1:< 10% surface area coverage; 2: between ≤ 10% and < 50%; and 3: ≥ 50%; Fig 1).

**Table 2:** List of scoring criteria for categorical reflex responses (i.e., absent, weak, moderate, and strong) of plaice (*Pleuronectes platessa*) in the order tested within 5 s of observation after stimulus.

Reflex	Stimulus	Absent	Weak	Moderate	Strong
Body flex	Fish is held outside the water on the palms of two hands (touching each other) with its belly facing up and its head and tail unsupported.	No active movement, the body rests limp on the hand.	Tail is moving slightly, but not beyond the plain of the hand.	Tail is flexing beyond the plain of the hand. Body may move—spastic flexion; or slowly slipping off the hand.	The fish is actively trying to move head and tail towards each other—curling reflex; or quickly slipping off the hand.
Righting	Fish is held underwater at the surface on the palms of two hands (touching each other) with its belly facing up and then slowly released.	Fish drifts and sinks passively to the bottom of the container.	Fish appears stunned, but rights itself very slowly.	Fish appears stunned, but starts to turn after delay. The rotation should be swift.	Fish actively and quickly turns underwater.
Head complex	The fish is held by its body out of water with its dorsal side facing up and its head and operculum observed for 5 s. The head is pointing away from the handler.	No movement of operculum and/or mouth.	Fish opens and closes its mouth and/or operculum just once.	Fish opens and closes its mouth and/or operculum more than once, but with a delay.	Fish immediately opens and closes its mouth and/or operculum more than once.
Evasion	Fish is held underwater at the surface in an upright position by supporting its belly with the fingers and holding its back by the thumbs. Then the thumbs are lifted and the fish released, while still supporting its belly by the fingers.	No active swimming movement; drifting motionless or swims at water surface.	Fish evades to the bottom, but swimming movement is weak.	Fish evades, but with a delay, and swimming movement is clear.	Fish immediately swims to the bottom.
Stabilise	This reflex is scored straight after evasion. No extra handling is required.	After evasion, the fish does not come to rest (keeps swimming or slides across the bottom of container).	Fish settles actively at the bottom, but shows no body and/or fin movement.	Fish actively settles at the bottom, and shows fin movement.	Fish actively settles at the bottom, and shows vigorous up-and-down body and/or fin movement.
Tail grab	The fish is being held between thumb and index finger.	Fish does not struggle free; it remains motionless upon release.	Fish does not struggle free; no swimming movement, but swims away upon release.	Fish does not struggle free, but moves its body as if it attempts to swim away.	The fish actively struggles free and swims away.

Intensity of a response increases from absent to strong. The speed of a response for weak and moderate categories may be delayed; for strong it should be immediate.

A seawater-filled, 30-L container (60 cm Length x 40 cm Width x 12 cm Height) placed on a table on-board the vessel, was used for all reflex and injury assessments. The person who handled the fish for the assessments stood in front of the container, one rater to the left and two raters to the right side, in 1-m proximity to each other and the container. After each gear deployment, raters rotated their observational positions to avoid any bias in scoring ability from an impaired field of view (i.e., a video camera tripod) or a skewed perspective.

Each fish was scored for the described six reflexes (Table 2; S1 Video) within 5 s of observation [6]. These reflexes were selected based on [6], with reflex actions, if present, being clearly visible to all raters who examined the fish without handling it themselves: (1) the body flex reflex, which was scored with the fish being held on the palm of a flat hand, with its ventral side facing up; (2) the righting reflex was scored by holding the fish on the palm of two hands, with its belly facing up and then released underwater; (3) the head complex involved the fish being held out of the water, and the mouth and operculum were observed for any movement; (4) evasion was scored when the fish was held at the water surface, with its dorsal side facing up and then gently released; (5) an attempt of a free-swimming fish to find a resting position on the bottom of the container was scored as the stabilise reflex; and (6) the tail grab reflex involved holding the fish by its tail between thumb and index finger.

Unlike for the injury assessment, scoring criteria for each reflex response were read out loud to the raters at the beginning of each trip (Table 2). Oral training was assisted by a pictorial guide sheet detailing response criteria at each threshold level per reflex (Table 2). No further discussion about nuances in criteria interpretation were allowed throughout the trip to keep observations independent. Following reflex assessment, each fish was examined for the severity of point bleeding and bruising to both the head and body on an categorical scale. Then, each fish was measured to the nearest cm (total length, TL) and tagged with a t-bar anchor tag following [6]. After tagging, fish were placed in a water-filled 15-L bucket. Once the bucket contained five fish, these were transferred to the on-board holding unit on-deck. This unit consisted of 30-L containers (60 cm L x 40 cm W x 12 cm H) stacked on top of each other as a flow-through system with ambient seawater (containers stacked 4 x 7).

### Mortality assessment

At the end of each trip, the on-board holding unit was transferred to the laboratory and fish from each 30-L container were released into 18 independent 124-L containers (75 cm L x 40 cm W x 30 cm H) connected to a recirculation system at ambient seawater temperature, as described by [35]. Plaice were offered defrosted brown shrimp (*Crangon crangon*) or ragworm (*Nereis virens*) as food after 7 d of monitoring. Fish were checked for mortality two times per day during the first week, and daily for the remainder of the second week of monitoring. Any food remains and/or dead fish were removed and the fish ID and time of mortality was noted. Throughout on-board and lab-based holding of fish, dissolved oxygen (mg/L) concentration, conductivity (PSU), and temperature (°C) of water-filled containers were monitored with a YSI Pro2030 handheld probe. IDs of survivors were noted at the end of the monitoring period.

### Data and analyses

To test for any inter-rater differences of categorical reflex and injury scores, binomial generalised mixed effects models (GLMMs) were fitted with the lme4 package [36] in the R language 3.2.5 (freely available from http://cran.stat.auckland.ac.nz/)). These GLMMs included as fixed effects rater, description (name of each reflex or injury type), and their interaction; and as random effect fish ID. For the binomial GLMMs, categorical reflex or injury scores were turned into binary variables (Fig 1). For the reflexes this was done by assigning the category ‘weak’ to either absent (0), to allow for the rule of doubt whereby weak responses may be scored as absent [7, 24–26], or as ‘strong’ (1), to allow weak reflex actions to be counted as a response (Fig 1). For injuries, absence equated to ‘0’, the categories ‘1’, ‘2’ and ‘3’ were classified as present (1). As another option, injury category 1 was classified as absent, considering that even a single point bleeding of an otherwise immaculate looking fish could have misled a rater to score it as present. Inter-rater agreement was quantified by computing Fleiss’ kappa using the irr package [37] in R. The stronger the agreement between raters is, the closer to 1 the value of kappa is. In case of perfect agreement, kappa equals 1, while when there is no agreement between raters kappa approaches zero [38].

To test for any inter-rater differences of the continuous scores, linear mixed-effects models (LMMs; lme4 package in R) [36] were analysed including the same fixed and random effect as for the categorical scores above. Inter-rater reliability was estimated by the performance of intra-class correlation (ICC), which was based in our case on the ratio of the variability among rater’s reflex scores over the sum of this variance plus error, thus ranging between 0 and 100% [39]. A higher value of ICC reflects a higher agreement among the raters for a given reflex type. The ICC measure of association was estimated using the psych package [40] in R.

To test for inter-rater differences of reflex (termed ‘R index’) and reflex & injury (‘R&I index’) indices, LMMs were used with rater as fixed effect and fish ID as random effect. Models were compared based on Akaike information criterion (AIC). ICC was used to test for inter-rater reliability. R indices were calculated per fish as 1 minus the mean score of impaired reflexes, and R&I indices as 1 minus the mean score of impaired reflexes and present injuries. Therefore, the values of injury scores were reversed to reflect that a reflex-responsive fish (reflex scores > 0) is more likely to be less injured (hence, injury scores > 0).

Prior to the calculation of reflex indices based on continuous data, scores were divided by 100. Calculating these indices based on the categorical data however, required assigning values to the intermediate weak reflexes or injury scores, because each successive transition between the ordinal categories might not represent the same difference in reflex or injury intensity [33, 41]. Therefore, arbitrary values of 0, 0.25, 0.33, 0.50, 0.66, 0.75 and 1 were assigned to the reflex category weak and for the ordinal injury category ‘1’ values of 0.5 and 0.66 and for the category ‘2’ values of 0.0 and 0.33 (Fig 1).

For significance testing of the different models, analysis of deviance (Type III Wald chi-square tests) and post-hoc pairwise comparisons of least-square means with a Tukey correction for multiple comparisons were performed. Inter-rater reliability estimates and model results derived from categorical and continuous input data, respectively, are difficult to compare with each other due to completely different variance structures.

To address our second objective of evaluating the effect of inter-rater differences of R and R&I indices and the type of scoring scale on the relationship of these indices with delayed mortality, non- and semi-parametric survival analyses (i.e. Kaplan-Meier, KM, survival curves and Cox proportional hazard models [coxph]) were used. Kaplan-Meier survival curves were visually compared among raters for segregation of survival rates in relation to discrete levels of R and R&I indices. Hazard ratios of the coxph models were compared to examine which rater’s survival curve produced the highest proportional hazard ratio. Additionally, the effect of fish size on (delayed) mortality was examined by using coxph. The model included survival as the response variable and the mean reflex index (based on either categorical or continuous scores) and the interaction of the respective score with fish size as explanatory variables. For the survival analyses, the survfit- and coxph-functions of the survival package [42] were used.

Further, logistic mixed regression models were used to model the influence of R (based on either categorical or continuous scores) and R&I indices (based on categorical scores) per rater and TL (as fixed effects) on mortality (the response variable) at 14 days after capture (in captivity). Candidate models (*n* = 35) were built, including all possible combinations of arbitrary values assigned to the intermediate categories of categorical reflex (i.e., weak) and injury scores (categories ‘1’ and ‘2’; Fig 1). All candidate models included as random effect batch ID (unique to a sampled batch of five fish nested within a trawl, nested within a trip). The most parsimonious model was validated by checking how reliably it would predict mortality events from one third of the randomly split data set. The level of significance was set at *p* < 0.01. All analyses were done in R.

## Results

During the four trips, the catch from 17 trawl deployments were sampled. Deployment duration varied between 30 and 50 min (43 ± 5 min, Mean ± SD, Table 1). Three hundred and four undersized plaice (21 ± 5 cm TL, Mean ± SD) were scored for reflex responses and injuries, and in total 912 (3 raters * 304 plaice) reflex and injuries assessments were completed. For the logistic regression analysis of the effect of inter-rater differences and type of scoring scale on the relationship between R and R&I indices with delayed mortality, 126 observations were excluded because, in those instances, raters failed to score the same reflex or injury within the 5 s timeframe.

### Reflex scores

**Fig 2:**
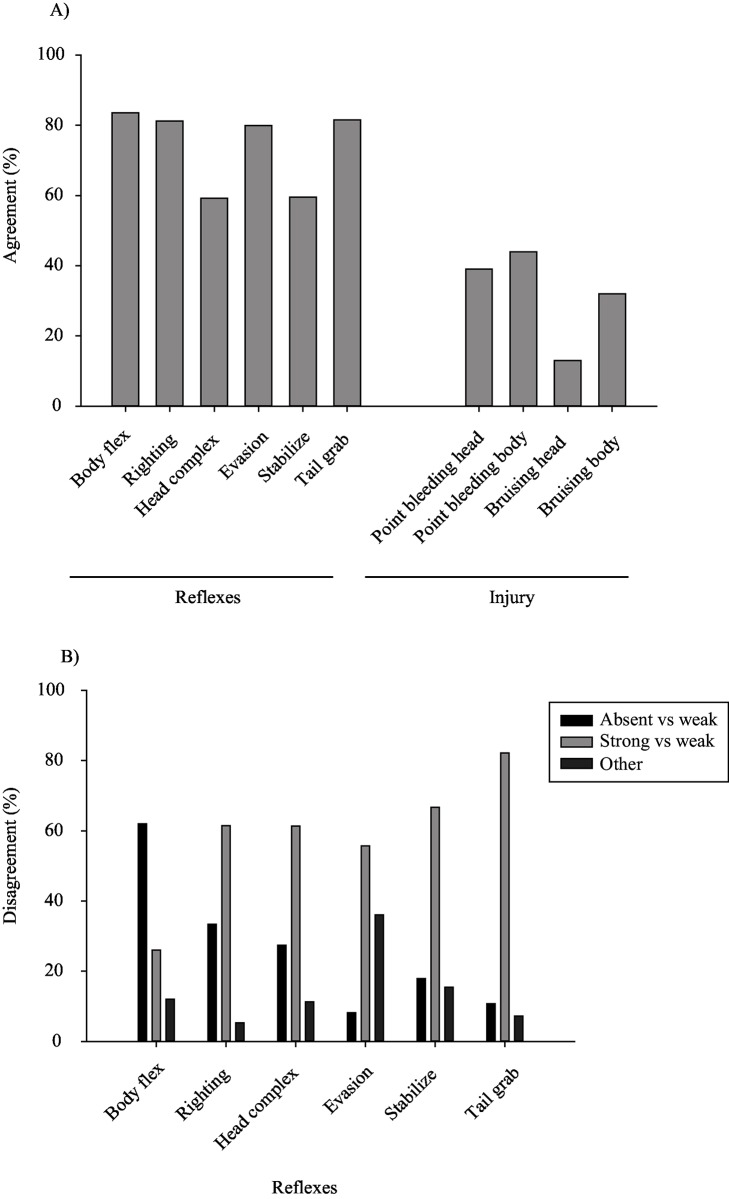
Rater (dis)agreement of categorical scores. (A) Proportion (%) of rater agreement between categorical reflex and injury scores. (B) Disagreement was apportioned into differences between the categories absent and weak (black), strong and weak (light grey) and other differences (such as between the categories absent and strong or every rater appointed another category; dark grey).

Of all categorical reflex scores (304 fish * 3 raters * 6 reflexes), about half were rated as strong (unimpaired, 53%), followed by absent (impaired, 30%), or weak (17%). Overall, 80% of the fish were scored exactly the same by all three raters for the body flex, righting, evasion, and tail grab reflexes (Fig 2A). When scores did not correspond the discrepancy was predominantly between weak and strong categories, except for the body flex where differences were between weak and absent (Fig 2B).

Agreement among rater reflex scores was higher than it was for injury. For categorical scores, agreement among rater’s reflex scores improved and differences became insignificant, when all weak reflex scores were considered as strong during data analysis (*p* > 0.1, GLMM; Table 3). In that case, higher levels of agreement occurred when scoring for righting and tail grab (0.88; based on Fleiss’ kappa), followed by body flex and evasion (0.76), head complex (0.67) and stabilise (0.66). In contrast, if ‘weak’ scores were included with ‘absent’, differences between raters became more prominent (significant interaction between reflex description and rater; Table 3). Accordingly Fleiss’ kappa values were consistently lower when compared to the above scenario: righting (0.76), followed by body flex (0.75), tail grab (0.69), evasion (0.63), head complex (0.59) and stabilise (0.55).

**Table 3:** Significance of variables and their interactions of a generalised linear mixed model (GLMM) based on Wald III Chi-square test results of categorical reflex and injury scores.

Definition of the Intermediate category	Reflexes/injuries	Variables	Chisq	Df	Pr(> Chisq)
As strong	Reflexes	Intercept	84.23	1	< 2e^-16^
		Description	388.90	5	< 2e^-16^
		Rater	0.55	2	0.76
		Description:Rater	12.33	10	0.26
As absent	Reflexes	Intercept	144.26	1	< 2.2e^-16^
		Description	334.76	5	< 2.2e^-16^
		Rater	4.04	2	0.13
		Description:Rater	40.46	10	1.39e^-06^
As present	Injuries	Intercept	154.28	1	< 2.2e^-16^
		Description	157.38	3	< 2.2e^-16^
		Rater	116.06	2	< 2.2e^-16^
		Description:Rater	37.53	6	1.39e^-06^
As absent	Injuries	Intercept	108.45	1	< 2.2e^-16^
		Description	79.58	3	< 2.2e^-16^
		Rater	38.43	2	4.52e^-09^
		Description:Rater	105.30	6	< 2.2e^-06^

For the binomial model, the intermediate categories (‘weak’ reflexes, or ‘1’ for injuries) were either defined as present (strong for reflexes) or absent, while the intermediate injury category ‘2’ was always defined as present.

When considering the continuous tVAS scale, reflex scores were always significantly different among raters (S1 Table). The reflex with the highest intra-class correlation was righting (intra-class correlation coefficient—ICC, 91%), followed by body flex and tail grab (88%), and then evasion (74%), head complex (73%) and stabilise (67%).

Pairwise comparisons revealed differences by rater as detected above for the more ambiguous reflexes, such as head complex and stabilise. Specifically, rater C stood out for these and also the tail grab reflexes, depending on whether weak was considered as strong or absent for categorical (S2 and S3 Tables, respectively), and also for continuous scores (S4 Table). Rater B scored the stabilise reflex differently compared with the other raters, for both categorical (if absent included weak), and continuous scales (S3 and S4 Tables). For the continuous scale, scores of head complex differed significantly among all raters (S4 Table).

### Injury scores

For injury, the majority of plaice were scored as being uninjured (score 0: 36%) or just slightly injured (score 1, <10% surface area covered by bleeding injury: 51%, score 2, ≤ 10% and < 50%: 10%, and score 3, ≥ 50%: 3%). Overall, agreement among raters was low (< 50%; Fig 2A). Most notably, scores for bruising to the head or body deviated among raters (Fleiss’ kappa of 0.27 and 0.21, respectively), when the intermediate category ‘1’ was classified as absent, and category ‘2’ as present). Inter-rater agreement was even lower when intermediate categories ‘1’ and ‘2’ were classified as present: for bruising to head and body (Fleiss kappa: 0.08 and 0.10, respectively).

When examining differences among raters, it was evident that all injury types were scored differently among all three raters, regardless whether category ‘1’ was classified as absent or present (Table 3; Fig 1A). When ‘1’ was assigned to strong, all raters scored differently for point bleeding body and bruising body, while scores from raters A for point bleeding head and bruising head stood out relative to the other two raters (S5 Table). When ‘1’ was assigned to absent, there was always one rater that scored differently relative to the other two. However, this was not done consistently across the different injury types. Rate C scored differently from the other raters for point bleeding on the head, rater A for bruising head, and rater B for point bleeding body and bruising body (S6 Table).

### Reflex and injury indices

Consistent differences between rater C and raters A and B in scoring individual reflexes (such as head complex, evasion, and tail grab) and injuries indicated significant disagreement among raters, when impaired reflex and present injury scores were averaged to calculate either reflex (R) or reflex & injury (R&I) indices. For example, reflex and reflex & injury indices based on categorical scores (R.cat and R&I.cat, respectively) differed significantly among raters (LMM, *p* <0.001; S7 and S8 Tables), regardless of what arbitrary value was assigned to the intermediate categories (weak for reflexes, and ‘1’ and ‘2’ for injuries). For the individual categorical reflexes rater C scores did not significantly differ from raters A and B (S9 Table), and agreement improved among raters when the weak and strong reflex category were pooled. For example, assigning a value of 0.66 to the weak reflex category, produced the most parsimonious model when fitted to the reflex index (LMM, AIC = -1182; S7 Table) and highest inter-rater agreement, ICC-value (87%).

The LMM fitted to the R & I index with a value of 0.66 assigned to the weak category (reflexes) and values of 0.66 and 0.33 to the categories ‘1’ and ‘2’ (injuries), respectively (Fig 1A), produced the most parsimonious model (LMM, AIC = -1659, S8 Table). Inter-rater agreement based on ICC was also highest (83%) for the R&I index with the above mentioned values assigned to the intermediate categories. R&I indices were significantly different among all three raters (S10 Table).

Based on continuous scores, reflex indices (R.con) were significantly different among raters (LMM, *p* < 0.001; S11 Table). The level of agreement among raters was 86% based on ICC. Pairwise comparisons of least-square means (lsmeans) of reflex indices showed these indices were completely different among all three raters (S12 Table).

### Relationships with delayed mortality

**Fig 3:**
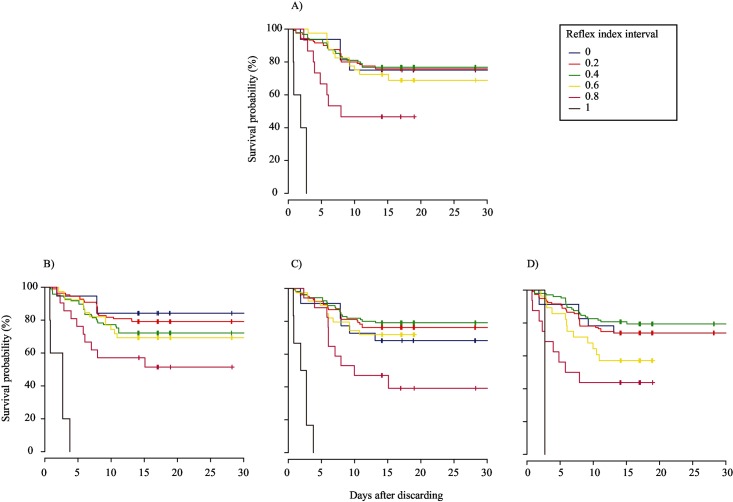
Non-parametric Kaplan–Meier survival probability estimates over days of monitoring of discarded plaice (*Pleuronectes platessa*) at 0.2 intervals of the reflex index based on categorical scores. To calculate the reflex index, the three categories were assigned values of 0, 0.66, and 1 for absent, weak, and strong, respectively. (A) mean; (B) rater A; (C) rater B; and (D) rater C.

**Fig 4:**
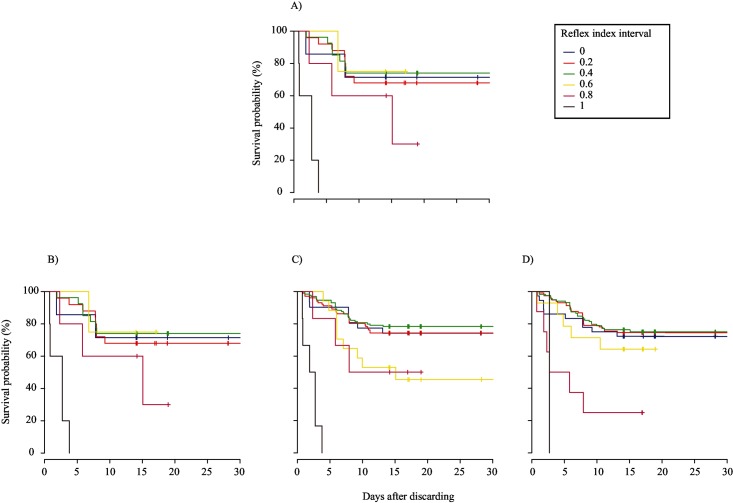
Non-parametric Kaplan–Meier survival probability estimates over 14 days of monitoring of discarded plaice (*Pleuronectes platessa*) at 0.2 intervals of the reflex index based on scores from the categorical scale. The three categories were assigned values of 0, 0, and 1 for absent, weak, and strong, respectively. (A) mean; (B) rater A; (C) rater B; and (D) rater C.

**Fig 5:**
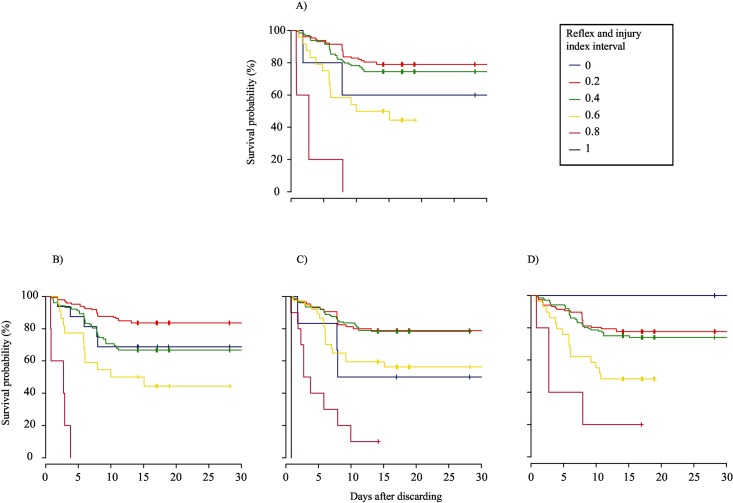
Non-parametric Kaplan–Meier survival probability estimates over 14 days of monitoring of discarded plaice (*Pleuronectes platessa*) at 0.2 intervals of the reflex & injury index based on scores from the categorical scale. The three reflex categories were assigned values of 0, 0.66 and 1 for absent, weak, and strong, respectively and the four injury categories were assigned values of 1, 0.66, 0.33 and 0 for ‘0’, ‘1’, ‘2’ and ‘3’, respectively. (A) mean; (B) rater A; (C) rater B; and (D) rater C.

**Fig 6:**
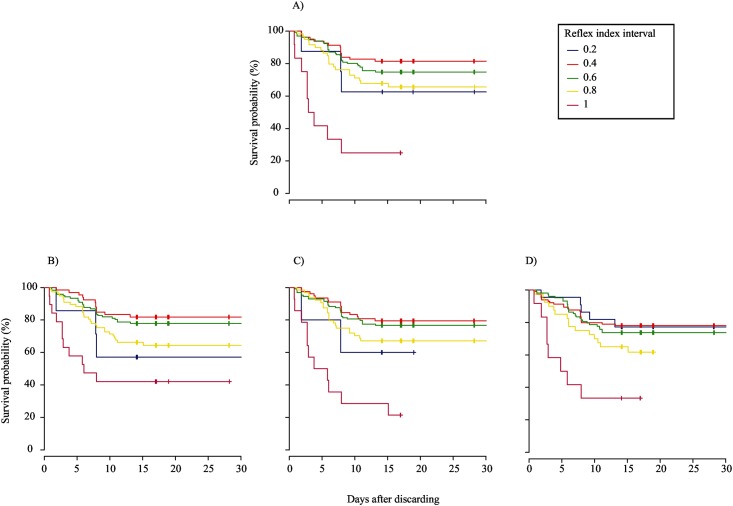
Non-parametric Kaplan–Meier survival probability estimates over 14 days of monitoring of discarded plaice (*Pleuronectes platessa*) at 0.2 intervals of the reflex index based on scores from the continuous scale. (A) mean; (B) rater A; (C) rater B; and (D) rater C.

The thirty controls tested on-board had limited impairment or injury (Mean R&I index ± SE = 0.07 ± 0.06), and all survived, in contrast with 50%, 75%, 94%, and 75% of treatment fish of trips 1 to 4, respectively. Based on visual inspection of the Kaplan-Meier survival curves, rater’s A categorical scores produced the best fit for the relationship between the R index and survival probability, when weak was assigned a value of 0.66, because at the end of the monitoring period each curve line was located above the curve line that represents the next greater interval score (Fig 3B). In contrast, when weak was combined with absent (0), the Kaplan-Meier curves intersected each other for all three raters (Fig 4). Compared to these fits, visual fit of the Kaplan-Meier plots of the R index (based on continuous scale) and R&I index was even better, because there was no overlap among raters of curve lines observed at the end of the monitoring period (Figs 5 and 6). The peculiar positions of the 0 (Fig 5B and 5C) and 0.2 curve lines (Fig 6B and 6C) can be ignored, because they were an artefact of the small sample size of fish being scored at this index interval. For both the R and R&I indices, rater A produced the lowest *p*-value and the highest concordance value (Table 4).

**Table 4:** Cox proportional hazard (coxph) regression model per rater with state of the fish (dead or alive) as response variable and the reflex (R) or reflex & injury (R&I) index as independent variables.

Scale	Variable	Rater	Coef	Exp (coef)	Se (coef)	z	Pr (>|z|)	Concordance	Rsquare
**Categorical**									
	R index	A	2.24	9.42	0.52	4.33	1.51e^-05^	0.61	0.058
		B	1.99	7.28	0.54	3.69	2.28e^-04^	0.57	0.043
		C	1.80	6.00	0.56	3.18	1.49e^-03^	0.57	0.032
	R&I index	A	4.58	97.33	0.72	6.37	1.90e^-10^	0.66	0.118
		B	3.31	27.25	0.76	4.37	1.20e^-05^	0.59	0.061
		C	3.59	36.24	0.80	4.50	6.80e^-06^	0.60	0.063
**Continuous**									
	R index	A	2.61	13.59	0.69	3.79	1.51e^-04^	0.62	0.049
		B	2.56	12.91	0.70	3.64	2.73e^-04^	0.60	0.045
		C	2.01	7.45	0.65	3.10	1.93e^-03^	0.58	0.032

**Fig 7:**
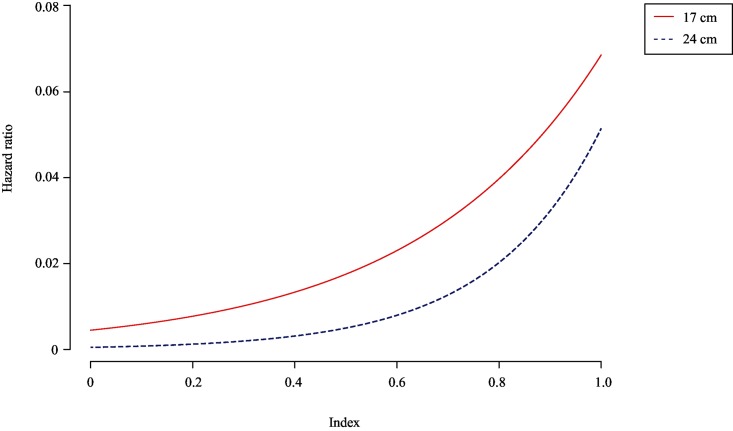
Relationship between the hazard ratio (coxph regression model for a mean rater with state of the fish as response variable and the reflex index based on continuous reflex scores; see S13 Table) and the reflex impairment index based on continuous scores for plaice (*Pleuronectes platessa*) at 17, and 24 cm in total length.

A coxph model was used to test whether fish size influenced reflex (and injury) scores and, thus, had an impact on the relationship with survival probability (S13 Table). The interaction between R and R&I indices and TL was always significant in its relation to survival (*p* < 0.001; S13 Table). This means that with increasing TL, the effect of vitality status becomes less relevant in predicting survival, or in other words the risk to die becomes less (Fig 7).

For the GLMM analysis, models with the lowest AIC, always and for each rater, included R&I over R indices. The lowest AIC included scores by rater A, where intermediate categories of reflexes were assigned a value of 0.75 and intermediate categories of injury values of 0.66 and 0. Fit improved when rater’s slight injuries (ordinal value ‘1’) were considered absent rather than present. However, for reflexes, model fit improved when rater A’s and B’s weak scores were considered as strong, whereas for rater C, a weak reflex was better classified as absent. For all raters, the most parsimonious models indicated a significant negative relationship between fish size and mortality (Table 5): the larger the fish, the lower probability of mortality. Whereas the relationship between the R&I index and mortality was significantly positive (Table 5). The higher the index, the higher probability of mortality. The optimal model including the R&I index per rater and TL as co-variates was able to correctly predict observed mortality in 81%, 77% and 69% of the cases when applied to the validation dataset for rater A, B, and C, respectively.

**Table 5:** Each rater’s most parsimonious logistic regression model for survival of European plaice (*Pleuronectes platessa*), with co-variates: Reflex impairment and injury index based on categorical scores (R&I.cat), and total length (TL).

Rater	Parameter	Estimate	SE	z-value	Pr(>|z|)
**A**	Intercept	-0.09832	0.93056	-0.106	0.91585
	R&I.cat	5.69246	1.37566	4.138	3.5e-05*
	TL	-0.13390	0.04660	-2.874	0.00406*
**B**	Intercept	-0.05306	0.87068	-0.061	0.95140
	R&I.cat	3.89867	1.19551	3.261	0.00111*
	TL	-0.11476	0.04365	-2.629	0.00856*
**C**	Intercept	-0.41957	0.97064	-0.432	0.66555
	R&I.cat	6.98719	1.60053	4.366	1.27e-05 *
	TL	-0.15350	0.04716	-3.255	0.00113*

*p < 0.01.

## Discussion

This is the first study to quantify inter-rater reliability of reflex impairment and injury scoring. Our results indicate that low cost reflex impairment and injury scoring can be considered a robust technique to evaluate lethal stress and damage of a fish intended for discard given that rater-based reflex and reflex & injury indices were always significantly associated with mortality probability, despite some inter-rater differences, especially among injury scores. Our results further corroborate widespread evidence that reflex impairment is a strong predictor of survival probability amongst discarded organisms [6–7, 24], and that the fit of impairment indices with survival can be improved, by incorporating injury scores to fine-tune predictions [6, 10].

Nevertheless, in the context of this study, it was demonstrated that differences in scores and the type of scoring scale (categorical vs continuous) do (slightly) affect the reflex action mortality predictor (RAMP) relationship with survival. Using a continuous scale led to significant differences among raters. Moreover, using a reflex index based on continuous scores did not improve model fits with mortality compared to the reflex & injury index based on categorical reflex scores. Nevertheless, all indices were confounded by fish size in their relationship with delayed mortality. The importance of the reflex and reflex and injury indices in association with survival probability diminished with increasing fish size. Larger flatfish might be energetically more resilient towards capture stress than smaller individuals [6].

Comparing the relative merit between using a categorical versus a continuous scale, introducing another, third category to describe the intensity of a present reflex response seemed more valuable than scoring with a continuous scale. Scoring a fish as more lively and less injured than it may have appeared (by giving intermediate reflex and injury categories more weight in assigning arbitrary values to these categories), did improve predictions of survival probability, a result confirming earlier observations [30]. The weak reflex category was valuable to minimise differences between rater’s scores, especially in this study, where coincidentally the majority of reflex responses were scored as present (possibly due to benign fishing practices), and doubt existed predominantly between weak and strong reflex responses.

Beyond the effect of an intermediate category, training and experience of raters seemed to have mattered to some extent despite not being tested as part of the original experimental design, as was already demonstrated in other animal welfare studies [43–45]. Although experience seemed less relevant if training was done, a lack of previous experience might still explain some of the observed deviations in reflex scores. Repeatedly, some of the categorical and continuous reflex scores of the least experienced rater (the student) were significantly different from the other two raters. However, disagreement between the more experienced raters arose as a consequence over discussions initiated by one of them in accepting modifications to the rating criteria of the stabilise reflex throughout the trips, and which might illustrate an effect of an expectation bias [46]. For example, rater B expected <15 cm plaice to exhibit a weak stabilize reflex if no displacement occurred. This was based on his own observations that smaller fish “stuck” to the bottom of the container without any rigorous body movement, whereas the other raters—by strictly keeping to the criteria at the time—would have scored such a response as an absent stabilise. This could indicate rater B might have scored with the modified criterion in mind, before it was actually agreed upon, and raters A and C caught up on it later on throughout the course of the study following some post-trip discussions. Notwithstanding the above, raters A and B might have been influenced by their experience of having scored with the rule of doubt in the past, but were instructed not to in this work.

Training is most effective if all scoring criteria and responses are clearly defined and unambiguous [47]. For example, sometimes doubt existed, especially for the stabilise and tail grab reflexes, whether a present response was weak or strong. From the discussions that were held amongst the raters at the end of each sampling trip, it was evident, that for head complex and stabilise reflex the descriptions for each of the scoring categories as outlined in Table 1 became somewhat more arbitrary than for other reflexes. For example, for the head complex, considering more frequent movements of operculum and/or mouth as a strong rather than weak response is arbitrary, and a measurement of a ventilation rate might have been a more appropriate measure for this reflex [48]. Unsurprisingly, for both reflexes, inter-rater reliability was relatively low, regardless whether they were scored with a categorical or continuous scale. In contrast, the righting, tail grab and body flex were unambiguous reflexes with high inter-rater reliability regardless of the scale used. Unambiguous rating criteria and their clear communication and training, are especially relevant for projects and protocols involving multiple raters with diverse experience backgrounds and who may not be in direct contact with each other [5].

While effective at addressing the question of inter-rater variability, there were potential biases. One of which was that reflexes were scored by a rater without actually handling and ‘feeling’ the fish. Although untested, we argue that the reflex responses we considered can equally be scored by external examination without actually handling the fish. In addition, while reflexes are ultimately and immediately measurable, injuries such as bruising can show up after assessment and may indicate an additional source of bias and disadvantage to using injury scoring after capture while still on-board. Finally, in our models, apart from the reflex and injury scores and the fish size, other variables were necessary to explain variability in mortality, which were not included as co-variates in our logistic regression models (e.g., gear type, temperature, or air exposure). The mortality rates we observed were within ranges of previous research under similar conditions. Fish were to a great extent unimpaired and uninjured, as a consequence of relatively short gear deployments. Despite a gradient of air exposure by consecutively scoring batches of fish that were standing on deck in dry baskets, air exposure was not a stressor that killed the fish and did result in neither an increase in immediate nor delayed mortality among the treatments.

The results from our study have immediate implications for future or on-going discard survival research in Europe. The RAMP method has been widely used as a proxy for delayed discard mortality [10, 21, 28], which has focused attention on the potential applications of RAMP in the context of the high survival exemption rule for the European landing obligation [6, 49]. Under this rule, a species may be discarded if it can be scientifically demonstrated that the probability to survive discarding is high [49]. To argue the case for an exemption, a robust, species-specific fleet-scale discard mortality estimate has to be produced that can stand criticism by a European expert panel [50]. To facilitate the generation of a fleet-scale discard mortality estimate, an established RAMP relationship between reflex impairment and/or injury, and mortality may be used to model survival probability of individuals for which only reflex impairment (and injury) scores were collected during at-sea monitoring campaigns [22, 51]. For this purpose, multiple observers (i.e., raters) may be employed who may collect reflex and injury scores across national data collection programmes. Such initiatives will benefit from these findings, which suggest that intra- and inter-reliability training be conducted, including resources such as a consensus atlas (pictograms and online video tutorials describing each reflex stimulus and response) as used for example in medical sciences [52].

## Supporting information

S1 Video Video clip detailing reflex assessments on-board. (WMV)

S1 Table Wald III Chi-square test results of a linear mixed model (LMM) fitted to continuous reflex scores. (DOCX)

S2 Table Post-hoc Tukey test results of pairwise comparisons between raters (A, B, and C) of least-square means (Lsmean ± SE) and lower and upper 95% confidence intervals (CI) of categorical reflex scores (‘weak’ included in the strong category). (DOCX)

S3 Table Post-hoc Tukey test results of pairwise comparisons between raters (A, B, and C) of least-square means (Lsmean ± SE) and lower and upper 95% confidence intervals (CI) of categorical reflex scores (‘weak’ included in the absent category.) (DOCX)

S4 Table Post-hoc Tukey test results of pairwise comparisons between raters (A, B, and C) of least-square means (Lsmean ± SE) and lower and upper 95% confidence intervals (CI) of continuous reflex scores. (DOCX)

S5 Table Post-hoc Tukey test results of pairwise comparisons between raters (A, B, and C) of least-square means (Lsmean ± SE) and lower and upper 95% confidence intervals (CI) of injury types (the intermediate categories ‘1’ and ‘2’ were assigned to present). (DOCX)

S6 Table Post-hoc Tukey test results of pairwise comparisons between raters (A, B, and C) of least-square means (Lsmean ± SE) and lower and upper 95% confidence intervals (CI) of injury types (the intermediate category ‘1’ was assigned to absent and ‘2’ to present). (DOCX)

S7 Table Wald III Chi-square test results of a linear mixed model (LMM) fitted to reflex indices (based on categorical scores with a value of 0.66 assigned to weak). (DOCX)

S8 Table Post-hoc Tukey test results of pairwise comparisons between raters (A, B, and C) of least-square means (Lsmean ± SE) and lower and upper 95% confidence intervals (CI) of reflex indices (based on categorical scores with a value of 0.66 assigned to a weak reflex reponse). (DOCX)

S9 Table Wald III Chi-square test results of a linear mixed model (LMM) fitted to reflex & injury indices (based on categorical scores with a value of 0.33 assigned to injury category ‘1’ and 0.66 assigned to a weak reflex and injury category ‘2’). (DOCX)

S10 Table Post-hoc Tukey test results of pairwise comparisons between raters (A, B, and C) of least-square means (Lsmean ± SE) and lower and upper 95% confidence intervals (CI) of reflex & injury indices (based on categorical scores). (DOCX)

S11 Table Wald III Chi-square test results of a linear mixed model (LMM) fitted to continuous reflex indices. (DOCX)

S12 Table Post-hoc Tukey test results of pairwise comparisons between raters (A, B, and C) of least-square means (Lsmean ± SE) and lower and upper 95% confidence intervals (CI) of continuous individual reflex indices. (DOCX)

S13 Table Cox proportional hazard (coxph) regression model for a mean rater with fish status (dead or alive) as response variable and the reflex index (based on categorical or continuous scores—R.cat or R.con, respectively) or reflex and injury (R&I) index and its interaction with fish size (TL) as independent variables. (DOCX)
