# Supplementary material for: Inter-rater reliability of categorical versus continuous scoring of fish vitality: Does it affect the utility of the reflex action mortality predictor (RAMP) approach?
Source: PLoS One. 2017 Jul 13;12(7):e0179092. doi: 10.1371/journal.pone.0179092 (PMC5509118; doi:10.1371/journal.pone.0179092)
Supplement: S1 Table — (DOCX) [file pone.0179092.s002.docx]

| **Variable** | **Chisq** | **Df** | **Pr(>Chisq)** |
| --- | --- | --- | --- |
| Intercept | 34.23 | 1 | 4.90e^-09^ |
| Description | 687.03 | 5 | <2.2e^-16^ |
| Rater | 1.75 | 2 | 0.42 |
| Description:rater | 137.21 | 10 | <2.2e^-16^ |
