# Supplementary material for: Inter-rater reliability of categorical versus continuous scoring of fish vitality: Does it affect the utility of the reflex action mortality predictor (RAMP) approach?
Source: PLoS One. 2017 Jul 13;12(7):e0179092. doi: 10.1371/journal.pone.0179092 (PMC5509118; doi:10.1371/journal.pone.0179092)
Supplement: S2 Table — (DOCX) [file pone.0179092.s003.docx]

| **Description** | **Rater** | **Lsmean** | **SE** | **Lower CI** | **Upper CI** | **Group** |
| --- | --- | --- | --- | --- | --- | --- |
| Body flex | A | -1.80 | 0.18 | -2.16 | -1.44 | 1 |
|  | B | -1.66 | 0.18 | -2.01 | -1.30 | 1 |
|  | C | -1.80 | 0.18 | -2.16 | -1.44 | 1 |
| Righting | A | 0.75 | 0.16 | 0.43 | 1.07 | 1 |
|  | B | 0.86 | 0.17 | 0.54 | 1.19 | 1 |
|  | C | 1.04 | 0.17 | 0.71 | 1.36 | 1 |
| Head complex | A | 1.59 | 0.18 | 1.24 | 1.94 | 1 |
|  | B | 1.58 | 0.18 | 1.23 | 1.92 | 1 |
|  | C | 2.21 | 0.20 | 1.81 | 2.60 | 2 |
| Evasion | A | 2.30 | 0.20 | 1.90 | 2.70 | 1 |
|  | B | 2.02 | 0.19 | 1.65 | 2.40 | 1 |
|  | C | 2.44 | 0.21 | 2.03 | 2.85 | 1 |
| Stabilize | A | 2.33 | 0.21 | 1.92 | 2.73 | 1 |
|  | B | 1.88 | 0.19 | 1.51 | 2.25 | 1 |
|  | C | 2.07 | 0.19 | 1.69 | 2.45 | 1 |
| Tail grab | A | 2.79 | 0.23 | 2.34 | 3.23 | 1 |
|  | B | 2.85 | 0.23 | 2.39 | 3.30 | 1 |
|  | C | 3.12 | 0.25 | 2.64 | 3.61 | 1 |

Significant differences were indicated by grouping raters in ascending order of Lsmeans.
