# Supplementary material for: Inter-rater reliability of categorical versus continuous scoring of fish vitality: Does it affect the utility of the reflex action mortality predictor (RAMP) approach?
Source: PLoS One. 2017 Jul 13;12(7):e0179092. doi: 10.1371/journal.pone.0179092 (PMC5509118; doi:10.1371/journal.pone.0179092)
Supplement: S3 Table — (DOCX) [file pone.0179092.s004.docx]

| **Description** | **Rater** | **Lsmean** | **SE** | **Lower CI** | **Upper CI** | **Group** |
| --- | --- | --- | --- | --- | --- | --- |
| Body flex | A | -2.97 | 0.24 | -3.43 | -2.50 | 1 |
|  | B | -2.54 | 0.21 | -2.96 | -2.13 | 1 |
|  | C | -3.12 | 0.25 | -3.61 | -2.63 | 1 |
| Righting | A | 0.22 | 0.15 | -0.08 | 0.52 | 1 |
|  | B | 0.32 | 0.15 | 0.02 | 0.62 | 1 |
|  | C | 0.45 | 0.15 | 0.15 | 0.76 | 1 |
| Head complex | A | -0.96 | 0.16 | -1.27 | -0.64 | 1 |
|  | B | -0.53 | 0.16 | -0.83 | -0.23 | 1 |
|  | C | -0.05 | 0.15 | -0.35 | 0.25 | 2 |
| Evasion | A | 1.38 | 0.17 | 1.05 | 1.71 | 1 |
|  | B | 1.42 | 0.17 | 1.09 | 1.75 | 1 |
|  | C | 2.12 | 0.19 | 1.74 | 2.50 | 2 |
| Stabilize | A | 0.06 | 0.15 | -0.24 | 0.36 | 1 |
|  | B | 0.72 | 0.15 | 0.41 | 1.03 | 2 |
|  | C | 0.26 | 0.15 | -0.04 | 0.55 | 1 |
| Tail grab | A | 1.23 | 0.17 | 0.91 | 1.55 | 1 |
|  | B | 1.24 | 0.17 | 0.92 | 1.57 | 1 |
|  | C | 2.17 | 0.19 | 1.79 | 2.55 | 2 |

Significant differences were indicated by grouping raters in ascending order of Lsmeans.
