# Supplementary material for: Inter-rater reliability of categorical versus continuous scoring of fish vitality: Does it affect the utility of the reflex action mortality predictor (RAMP) approach?
Source: PLoS One. 2017 Jul 13;12(7):e0179092. doi: 10.1371/journal.pone.0179092 (PMC5509118; doi:10.1371/journal.pone.0179092)
Supplement: S4 Table — (DOCX) [file pone.0179092.s005.docx]

| **Description** | **Rater** | **Lsmean** | **SE** | **Lower CI** | **Upper CI** | **Group** |
| --- | --- | --- | --- | --- | --- | --- |
| Body flex | A | 0.75 | 0.17 | 0.42 | 1.08 | 1 |
|  | B | 0.99 | 0.17 | 0.66 | 1.32 | 1 |
|  | C | 0.76 | 0.17 | 0.43 | 1.09 | 1 |
| Righting | A | 4.08 | 0.17 | 3.75 | 4.41 | 1 |
|  | B | 3.97 | 0.17 | 3.64 | 4.30 | 1 |
|  | C | 4.75 | 0.17 | 4.41 | 5.08 | 2 |
| Head complex | A | 2.58 | 0.17 | 2.25 | 2.91 | 1 |
|  | B | 3.24 | 0.17 | 2.91 | 3.57 | 2 |
|  | C | 4.35 | 0.17 | 4.02 | 4.68 | 3 |
| Evasion | A | 5.46 | 0.17 | 5.13 | 5.79 | 1 |
|  | B | 5.55 | 0.17 | 5.22 | 5.89 | 1 |
|  | C | 7.04 | 0.17 | 6.71 | 7.37 | 2 |
| Stabilize | A | 4.00 | 0.17 | 3.66 | 4.33 | 1 |
|  | B | 4.92 | 0.17 | 4.58 | 5.25 | 2 |
|  | C | 4.17 | 0.17 | 3.84 | 4.50 | 1 |
| Tail grab | A | 5.62 | 0.17 | 5.29 | 5.95 | 1 |
|  | B | 5.25 | 0.17 | 4.92 | 5.58 | 1 |
|  | C | 6.95 | 0.17 | 6.62 | 7.28 | 2 |

Significant differences were indicated by grouping raters in ascending order of Lsmeans.
