# Supplementary material for: Inter-rater reliability of categorical versus continuous scoring of fish vitality: Does it affect the utility of the reflex action mortality predictor (RAMP) approach?
Source: PLoS One. 2017 Jul 13;12(7):e0179092. doi: 10.1371/journal.pone.0179092 (PMC5509118; doi:10.1371/journal.pone.0179092)
Supplement: S5 Table — (DOCX) [file pone.0179092.s006.docx]

| **Description** | **Rater** | **Lsmean** | **SE** | **Lower CI** | **Upper CI** | **Group** |
| --- | --- | --- | --- | --- | --- | --- |
| Point head | A | 0.48 | 0.15 | 0.19 | 0.77 | 1 |
|  | B | 3.19 | 0.26 | 2.69 | 3.70 | 3 |
|  | C | 2.12 | 0.19 | 1.75 | 2.49 | 2 |
| Point body | A | 0.30 | 0.15 | 0.01 | 0.59 | 1 |
|  | B | 2.74 | 0.22 | 2.30 | 3.18 | 2 |
|  | C | 2.59 | 0.21 | 2.17 | 3.01 | 2 |
| Bruising head | A | -1.20 | 0.16 | -1.51 | -0.89 | 1 |
|  | B | 1.46 | 0.17 | 1.14 | 1.79 | 2 |
|  | C | 1.96 | 0.18 | 1.60 | 2.32 | 2 |
| Bruising body | A | -2.35 | 0.20 | -2.75 | -1.96 | 1 |
|  | B | 0.31 | 0.15 | 0.02 | 0.60 | 3 |
|  | C | -0.54 | 0.15 | -0.83 | -0.25 | 2 |

Significant differences were indicated by grouping raters in ascending order of Lsmeans.
