# Supplementary material for: Inter-rater reliability of categorical versus continuous scoring of fish vitality: Does it affect the utility of the reflex action mortality predictor (RAMP) approach?
Source: PLoS One. 2017 Jul 13;12(7):e0179092. doi: 10.1371/journal.pone.0179092 (PMC5509118; doi:10.1371/journal.pone.0179092)
Supplement: S6 Table — (DOCX) [file pone.0179092.s007.docx]

| **Description** | **Rater** | **Lsmean** | **SE** | **Lower CI** | **Upper CI** | **Group** |
| --- | --- | --- | --- | --- | --- | --- |
| Point head | A | -2.84 | 0.24 | -3.32 | -2.37 | 2 |
|  | B | -2.32 | 0.22 | -2.76 | -1.89 | 2 |
|  | C | -5.26 | 0.47 | -6.18 | -4.34 | 1 |
| Point body | A | -4.01 | 0.32 | -4.63 | -3.39 | 1 |
|  | B | -3.15 | 0.26 | -3.65 | -2.64 | 2 |
|  | C | -4.42 | 0.35 | -5.12 | -3.72 | 1 |
| Bruising head | A | -3.66 | 0.29 | -4.22 | -3.09 | 1 |
|  | B | -0.97 | 0.19 | -1.34 | -0.60 | 2 |
|  | C | -0.89 | 0.19 | -1.26 | -0.53 | 2 |
| Bruising body | A | -4.26 | 0.34 | -4.93 | -3.60 | 1 |
|  | B | -2.40 | 0.23 | -2.84 | -1.95 | 2 |
|  | C | -3.40 | 0.32 | -4.62 | -3.38 | 1 |

Significant differences were indicated by grouping raters in ascending order of Lsmeans.
