# Supplementary material for: Inter-rater reliability of categorical versus continuous scoring of fish vitality: Does it affect the utility of the reflex action mortality predictor (RAMP) approach?
Source: PLoS One. 2017 Jul 13;12(7):e0179092. doi: 10.1371/journal.pone.0179092 (PMC5509118; doi:10.1371/journal.pone.0179092)
Supplement: S9 Table — (DOCX) [file pone.0179092.s010.docx]

| **Variable** | **Chisq** | **Df** | **Pr(>Chisq)** |
| --- | --- | --- | --- |
| Intercept | 1793.94 | 1 | <2.2e^-16^ |
| Rater | 200.52 | 2 | <2.2e^-16^ |
