# Supplementary material for: Inter-rater reliability of categorical versus continuous scoring of fish vitality: Does it affect the utility of the reflex action mortality predictor (RAMP) approach?
Source: PLoS One. 2017 Jul 13;12(7):e0179092. doi: 10.1371/journal.pone.0179092 (PMC5509118; doi:10.1371/journal.pone.0179092)
Supplement: S12 Table — (DOCX) [file pone.0179092.s013.docx]

| **Rater** | **Lsmean** | **SE** | **df** | **Lower CI** | **Upper CI** | **Group** |
| --- | --- | --- | --- | --- | --- | --- |
| A | 0.62 | 0.01 | 363.43 | 0.60 | 0.64 | 3 |
| B | 0.60 | 0.01 | 367.89 | 0.58 | 0.62 | 2 |
| C | 0.53 | 0.01 | 367.43 | 0.51 | 0.55 | 1 |

Significant differences were indicated by grouping raters in ascending order of Lsmeans.
