# Supplementary material for: Inter-rater reliability of categorical versus continuous scoring of fish vitality: Does it affect the utility of the reflex action mortality predictor (RAMP) approach?
Source: PLoS One. 2017 Jul 13;12(7):e0179092. doi: 10.1371/journal.pone.0179092 (PMC5509118; doi:10.1371/journal.pone.0179092)
Supplement: S13 Table — (DOCX) [file pone.0179092.s014.docx]

| Variable | Coef | Exp (coef) | Se (coef) | z | Pr (>\|z\|) | Concor-dance | R-square |
| --- | --- | --- | --- | --- | --- | --- | --- |
| Reflex index (cat) | 6.37 | 583.29 | 1.11 | 5.73 | 9.90e^-09^ | 0.66 | 0.094 |
| Rcat : TL | -0.19 | 0.83 | 0.05 | -3.93 | 8.55e^-05^ |  |  |
| Rcon | 6.34 | 567.29 | 1.06 | 6.00 | 1.97e^-09^ | 0.68 | 0.104 |
| Rcon : TL | -0.16 | 0.85 | 0.04 | -4.36 | 1.28e^-05^ |  |  |
| R&I | 1.11e^01^ | 6.88e^04^ | 1.46e^00^ | 7.61 | 2.80e^-14^ | 0.70 | 0.160 |
| R&I : TL | -2.86e^-01^ | 7.51e^-01^ | 5.64e^-02^ | -5.07 | 4.09e^-07^ |  |  |
